# Rising from the Sea: Correlations between Sulfated Polysaccharides and Salinity in Plants

**DOI:** 10.1371/journal.pone.0018862

**Published:** 2011-04-28

**Authors:** Rafael S. Aquino, Clicia Grativol, Paulo A. S. Mourão

**Affiliations:** Laboratório de Tecido Conjuntivo, Instituto de Bioquímica Médica and Hospital Universitário Clementino Fraga Filho, Universidade Federal do Rio de Janeiro, Rio de Janeiro, Rio de Janeiro, Brazil; University of Leeds, United Kingdom

## Abstract

High salinity soils inhibit crop production worldwide and represent a serious agricultural problem. To meet our ever-increasing demand for food, it is essential to understand and engineer salt-resistant crops. In this study, we evaluated the occurrence and function of sulfated polysaccharides in plants. Although ubiquitously present in marine algae, the presence of sulfated polysaccharides among the species tested was restricted to halophytes, suggesting a possible correlation with salt stress or resistance. To test this hypothesis, sulfated polysaccharides from plants artificially and naturally exposed to different salinities were analyzed. Our results revealed that the sulfated polysaccharide concentration, as well as the degree to which these compounds were sulfated in halophytic species, were positively correlated with salinity. We found that sulfated polysaccharides produced by *Ruppia maritima* Loisel disappeared when the plant was cultivated in the absence of salt. However, subjecting the glycophyte *Oryza sativa* Linnaeus to salt stress did not induce the biosynthesis of sulfated polysaccharides but increased the concentration of the carboxylated polysaccharides; this finding suggests that negatively charged cell wall polysaccharides might play a role in coping with salt stress. These data suggest that the presence of sulfated polysaccharides in plants is an adaptation to high salt environments, which may have been conserved during plant evolution from marine green algae. Our results address a practical biological concept; additionally, we suggest future strategies that may be beneficial when engineering salt-resistant crops.

## Introduction

More than 6% of the world's land area consists of soil that is high in salinity, which is a critical agricultural problem that inhibits crop production worldwide [1]. High-salinity soil affects up to 20% of irrigated agricultural land, causing the permanent loss of 1.5 million hectares per year [2]. This situation is particularly worrisome considering almost one billion people worldwide are chronically undernourished, a number that continually increases with population growth [1]. The reality of an increased demand for food and a decrease in usable land due to high-salinity soils makes the development of salt-resistant plants critically important.

Extensive studies have been undertaken to understand the mechanisms involved in plant salt-tolerance; however, no salt-resistant crops have been engineered [3] [4] [5] [6]. Here, we suggest that marine algae, a salt-resistant ancestor of terrestrial plants, may be useful in understanding overall salt-tolerance mechanisms. Sodium exclusion, which is the major salt-resistance mechanism in plants, has recently been reported to also occur in marine algae [7] [8]. We therefore hypothesized that plants and algae may share other strategies to counteract salt stress.

An important difference observed when comparing algal and plant cell physiology is the presence of sulfated polysaccharides. While every type of marine algae studied to date produces at least one type of sulfated polysaccharide [9], these compounds are apparently absent in plants, suggesting a possible correlation with salt stress. Examples of sulfated polysaccharides produced by algae include the following: sulfated galactan produced by red algae (Rhodophyta) [10], the sulfated fucans (also known as fucoidans) produced by brown algae (Phaeophyta) [11], and the sulfated glucans, sulfated galactans, and sulfated arabinogalactans produced by green algae (Chlorophyta) [12] [13], which is the ancestor of higher plants [14]. Although the presence of sulfated polysaccharides in algae has been extensively studied, their physiological function remains unclear.

We recently identified the first sulfated polysaccharides in plants [15]: sulfated galactan in the cell wall of the marine angiosperm *R. maritima* Loisel [15]. The presence of sulfated polysaccharides in marine algae as well as in a salt resistant plant (halophyte) suggests a possible correlation between the presence of these compounds and salt tolerance; salt intolerant plants (glycophytes) do not contain these compounds.

In this study, plants were naturally or artificially exposed to different salinities, revealing that the concentration of sulfated polysaccharides, and the degree to which they were sulfated, correlated with the concentration of salt in the environment. Additionally, a survey that analyzed different plant species for the presence of sulfated polysaccharides demonstrated that halophytes, but not glycophytes, contained sulfated polysaccharides. Our results suggest that the occurrence of sulfated polysaccharides in higher plants is an adaptation to life in environments with high concentrations of salt.

## Results

### The concentration of sulfated galactan in *R. maritima* cell walls correlates with environmental salinity

**Figure 1:**
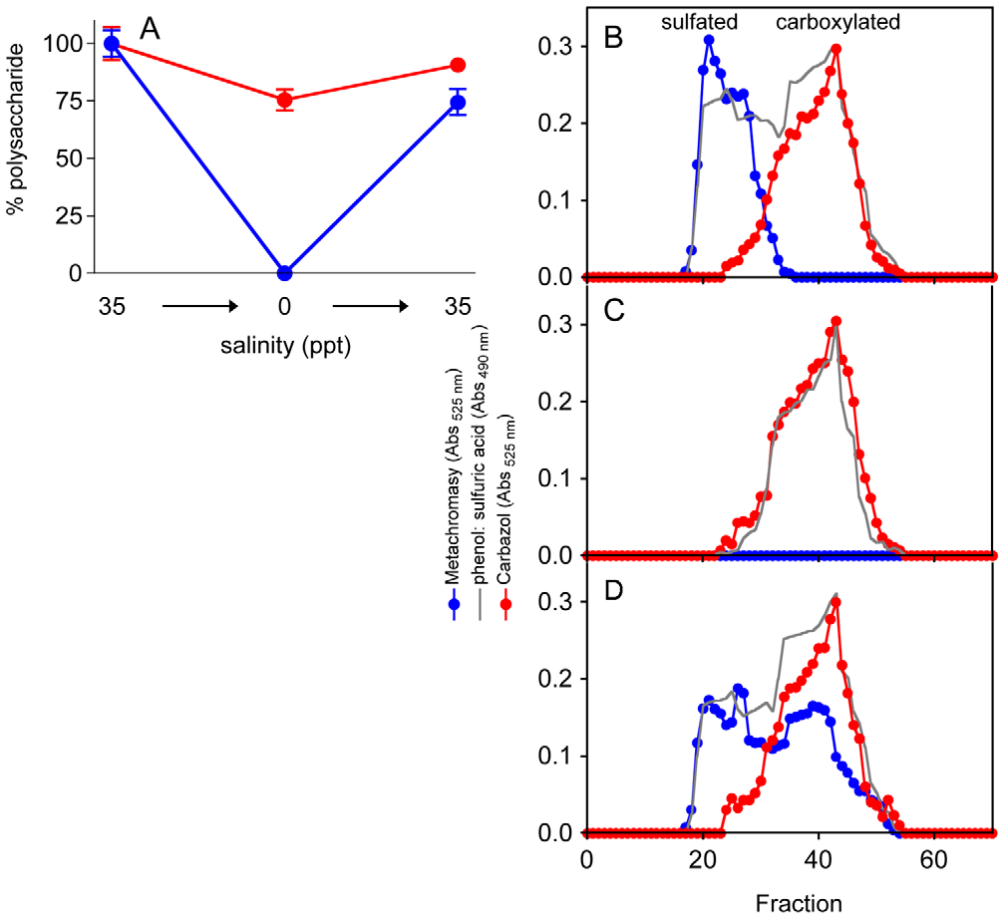
The biosynthesis of sulfated polysaccharides by *R. maritima* is correlated with salinity levels. Sulfated and carboxylated polysaccharides extracted from *R. maritima* were quantified (A) and analyzed by size exclusion chromatography (B–D). (A) Sulfated polysaccharides were quantified using a metachromatic reaction (solid circle), and carboxylated polysaccharides were quantified based on their hexuronic acid concentration using the carbazol reaction (open circle). Values are expressed based on their concentration at 35 ppt salinity (100%). Polysaccharides extracted from plants cultivated at 35 ppt salinity (B), after salt removal (C), and after salt was re-introduced (D) were analyzed by size exclusion chromatography on a Superose 12 column coupled to a FPLC system. Fractions were tested using the metachromasy reaction (solid circle), carbazol reaction (open circles), and phenol-sulfuric acid (continuous line) to quantify total hexose.

To determine if salinity affected the biosynthesis of sulfated polysaccharides, we analyzed the seagrass *R. maritima* after cultivation in varying salt concentrations. *R. maritima* is resistant to high levels of salt, surviving at salinities ranging from 0 to 70 ppt [16] [17]. Plant samples were collected and cultivated in an aquarium where the salinity was gradually decreased to freshwater salt concentrations (less than 0.5 ppt), grown for two weeks, and moved back to original conditions (35 ppt; average seawater salinity). Interestingly, sulfated galactans completely disappeared when plants were cultivated in the absence of salt (Fig. S1-A). The absence of sulfated polysaccharides was confirmed by agarose gel electrophoresis stained with toluidine blue and sulfate quantification using the barium assays (Fig. 1). The sulfated galactans concentration returned to levels found in the control samples after the salinity was restored. Carboxylated polysaccharides were only slightly affected by the variations in salinity (Fig. 1-A). These results were confirmed by subjecting the extracted polysaccharides to size exclusion chromatography, where in the absence of salt no sulfated galactan was observed in the sample (the fraction with higher molecular weight and positive for metachromatic reaction; Figs. 1-B and C). Curiously, samples obtained after the salinity was restored (Fig. 1-D) had two different fractions of sulfated galactans, one with a higher molecular weight, similar to the compound observed in control samples (Fig. 1-B), and a second fraction with a lower molecular weight. This result suggests that the sulfated galactans produced by *R. maritima* have a low-molecular-weight biosynthetic precursor. The sulfated galactans extracted from plants after the salinity was restored were analyzed using anion-exchange chromatography, which revealed a higher proportion of sulfated galactans that were sulfated to a lower degree. These compounds were eluted in lower salt concentrations (Fig. S2-A, peaks 1 and 2) when compared to the control. In addition, the polysaccharides containing fewer sulfate substitutions were found to incorporate sulfate earlier, which was observed after the seagrass was incubated with radioactive sulfate (Fig. S2-A). Agarose electrophoresis and autoradiography confirmed this result (Fig. S2-B and C). These results suggested that sulfated galactans produced by *R. maritima* have a biosynthetic precursor with a lower molecular weight that contains fewer sulfate substitutions. Additional structural studies will be necessary to understand in details the biosynthesis of sulfated galactans.

These results also suggest that the presence of sulfated galactans in the seagrass *R. maritima* is an adaptation to survival at high salinities. This hypothesis raises the question as to if other halophytes use the same strategy to survive in environments with high salinity.

### Sulfated polysaccharides are widespread among halophytes

**Figure 2:**
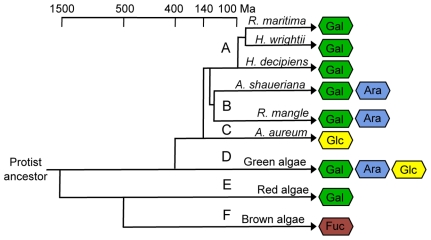
The occurrence of sulfated polysaccharides in plants and algae. Phylogenetic relationships, including the divergence times of algae, pteridophytes, angiosperms, and a summary of the monosaccharide composition of their sulfated polysaccharide are listed. Sulfated polysaccharides extracted from different plant species were purified using anion-exchange chromatography and their monosaccharide composition was evaluated by paper chromatography after acid hydrolysis. The sulfated polysaccharides from the seagrasses species *R. maritima*, *H. decipiens* and *H. wrightii* contain galactose [15] (A), those from the mangrove species *R. mangle* and *A. schaueriana* contain arabinose and galactose (B), while pteridophytes, which were represented by the species *A. aureum*, contain glucose (D). No sulfated polysaccharides were detected in terrestrial plants (*Z. maize*, *P. vulgaris* and *O. sativa*). Marine green algae (Chlorophyceae) contained a complex mixture of sulfated polysaccharides, with fractions containing arabinose, galactose and glucose [12] [13] (E). Red algae [10] (Rhodophyceae) (F) and brown algae [11] (Phaeophyta) (G) sulfated polysaccharides are composed mainly by galactose and fucose units, respectively.

A survey of halophytes revealed that the presence of sulfated polysaccharides in the plant kingdom is more common than previously thought. Polysaccharides were extracted from the root systems and leaves of different plants, purified by anion-exchange chromatography, and subjected to a monosaccharide analysis. A phylogenetic comparison between monosaccharide units of sulfated polysaccharides from plants (this work) and algae (references: [10] [11] [12] [13]) is shown in Figure 2. The seagrass species *Halophila decipiens* Ostenfeld, *Halodule wrightii* Aschers, and *R. maritima* possess sulfated galactans (Fig. 2-A), as previously described [15]. To determine if the presence of sulfated polysaccharides is exclusive to this group of “recently” evolved marine angiosperms, our survey included mangrove angiosperms. Our analyses of mangrove angiosperms indicated that *Avicennia schaueriana* Stapf & Leechman and *Rhizophora mangle* Linnaeus contained sulfated arabinogalactans (Fig 2-B), suggesting that the presence of sulfated polysaccharides in angiosperms may be a common feature of all halophytic aquatic plants. Going further back in the evolution of vascular plants, we investigated the presence of sulfated polysaccharides in a pteridophyte, an early evolved vascular terrestrial plant. The salt-resistant pteridophyte species *Acrostichum aureum* Linnaeus also possessed a sulfated polysaccharide, more precisely a sulfated glucan (Fig. 2-D). Additionally, we performed nuclear magnetic resonance (NMR) analyses on the purified samples to determine their chemical structures; however, the ^1^H NMR spectra at 600 MHz had broad, poorly resolved signals (Fig. S3), indicating the presence of heterogeneous chemical structures. Sulfated polysaccharides were not observed in the three species of glycophytes analyzed (*Zea mays* Linnaeus, *Oryza sativa* Linnaeus, and *Phaseolus vulgaris* Linnaeus). This brief survey suggests that the presence of sulfated polysaccharides is a common characteristic among halophytes with different evolutionary origins, but not glycophytes.

### Plants collected from environments with different salinities produce distinct sulfated polysaccharides

Further evidence for the correlation between salt concentration and sulfated polysaccharide biosynthesis was obtained using plants that were naturally exposed to different salinities. The sulfated polysaccharide concentration in the root system and leaves from all specimens collected was quantified (Table 1). Sulfated galactan concentrations decreased ∼70% in the root system of *R. maritima* when grown at lower salt concentrations (15 ppt), as compared to plants grown in a salinity of 35 ppt, suggesting that the sulfated polysaccharide content also correlates with salinity in nature. In addition, *H. wrightii* specimens grown in environments with relatively small differences in salinity (35 and 38 ppt) contained sulfated polysaccharides that differed in concentration by almost 50%.

**Table 1:** The concentration of sulfated polysaccharides from plants collected from environments with distinct salinities.

Plant	Species	Salinity (ppt)	Tissue	Sulfated polysaccharide (µg/mg dry weight)
Marine angiosperm	*R. maritima*	35	Leaf	7.2 (±1.7)
			Root	13.3 (±2.1)
		15	Leaf	4.7 (±0.7)
			Root	4.0 (±0.8)
	*H. wrightii*	38	Leaf	22.4 (±1.7)
			Root	10.0 (±1.3)
		35	Leaf	12.2 (±3.0)
			Root	6.6 (±1.1)
	*H. decipiens*	35	Leaf	5.15 (±1.3)
			Root	7.69 (±0.7)
Mangrove angiosperm	*A. shaueriana*	35	Leaf	16.7 (±0.7)
			Root	8.7 (±1.1)
	*R. mangle*	35	Leaf	18.0 (±2.1)
			Root	15.8 (±1.9)
Terrestrial angiosperm	*Z. mays*	0	Leaf	<0,001
			Root	<0,001
	*P. vulgaris*	0	Leaf	<0,001
			Root	<0,001
	*O. sativa*	0	Leaf	<0,001
			Root	<0,001
Mangrove pteridophyte	*A. aureum*	12	Leaf	<0,001
			Root	0.85 (±0.2)

**Figure 3:**
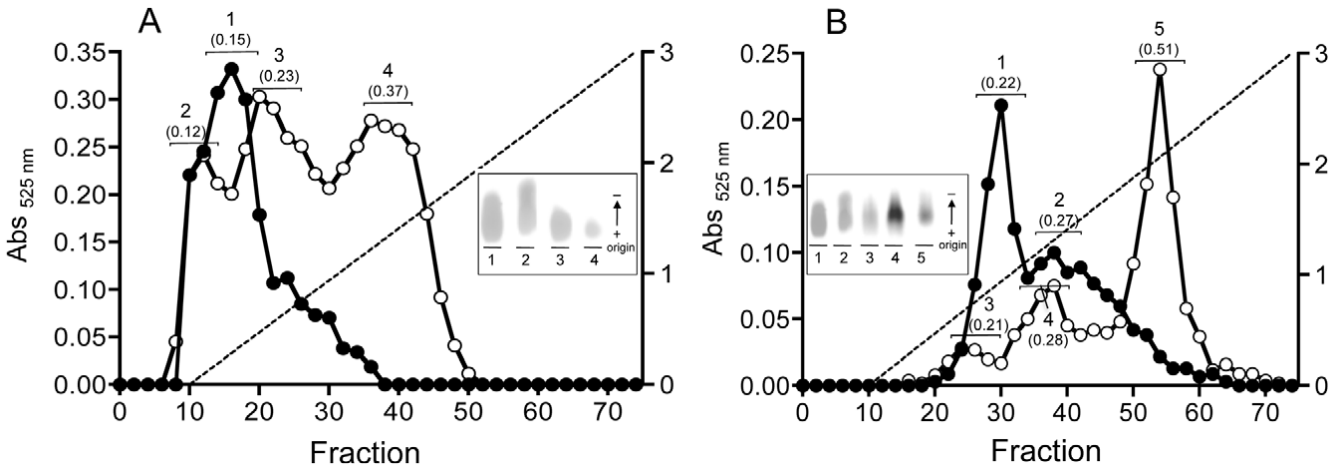
Salinity affects the degree to which sulfated polysaccharides are sulfated. Sulfated polysaccharides extracted from *H. wrightii* collected from an environment with 35 ppt salinity (A, closed symbol) and from a 38 ppt salinity environment (A, open symbol) were analyzed using anion-exchange chromatography (Mono-Q column coupled with a FPLC system). Sulfated polysaccharides from *R. maritima* collected from environments with 15 ppt (B, closed symbol) and 35 ppt (B, open symbol) salinity were also compared by the same procedure. Fractions were assayed using metachromasy. Some fractions were pooled, as indicated (1–5), and analyzed by agarose gel electrophoresis (graph inset), and sulfate concentration was determined (localized above fractions number, under brackets, and expressed as sulfate/monosaccharide units).

An anion-exchange chromatography analysis of the sulfated galactans produced by *R. maritima* and *H. wrightii* from habitats with different salinities suggested that sulfated galactans with distinct compositions were present. Instead of a single fraction that eluted with low concentrations of NaCl, as observed in *H. wrightii* grown in normal salt concentrations (35 ppt), the sample collected from higher salinity conditions had two additional fractions with a higher degree of sulfation (Fig. 3-A, peaks 3 and 4). On the other hand, *R. maritima* samples obtained from an environment with a lower salinity (15 ppt) possessed higher proportions of sulfated galactans with lower degree of sulfation when compared to the control (Fig. 3-B). Sulfate content was measured using the barium assay and expressed as sulfate units per monosaccharide (shown in brackets under the fraction numbers in Fig. 3). These results suggest that environments with high salinity not only increase the concentration of sulfated galactans in these plants but also affect the degree to which these compounds are sulfated.

### A correlation between salinity and carboxylated polysaccharide concentration was observed in the glycophyte *O. sativa*

**Figure 4:**
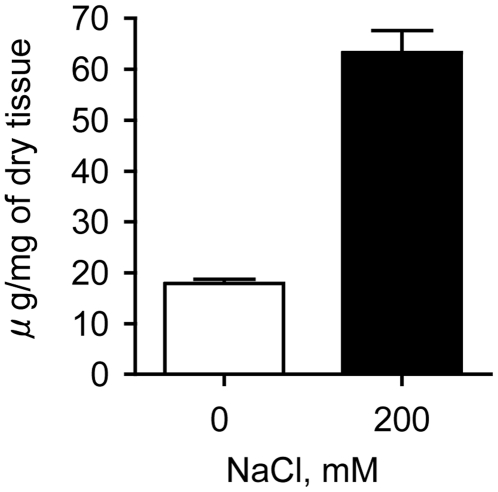
An increase in carboxylated polysaccharides is observed when rice is grown under salt stress. *O. sativa* specimens were grown for two weeks in freshwater (control) or in the presence of 200 mM NaCl. After polysaccharide extraction, the concentration of sulfated polysaccharides was determined by metachromasy (negative result, data now shown), and the presence of carboxylated polysaccharides was determined using the carbazol reaction.

Because we observed that the biosynthesis of sulfated polysaccharides was completely abolished when *R. maritima* was cultivated in the absence of salt, we decided to investigate if glycophytes can synthesize sulfated polysaccharides in the presence of high salt concentrations. To test this hypothesis, rice (*O. sativa*) was cultivated in the presence or absence of 200 mM of NaCl. Sulfated polysaccharides were not detected in either case, suggesting that high salt concentrations do not induce the synthesis of sulfated polysaccharides in rice. However, the concentration of carboxylated polysaccharides increased more than threefold in the plants cultivated in the presence of salt (Fig. 4), suggesting that acidic polysaccharides are important during growth in high salt environments.

### Discussion

In this study, we report that halophytes with diverse evolutionary origins possess sulfated polysaccharides, while the glycophytes tested did not. Analyses of plants exposed to diverse salinity conditions vary in terms of the amount of sulfated polysaccharides produced and in the degree to which those compounds are sulfated. These findings suggest a correlation between salinity levels and the production of these compounds. Overall, our results suggest that the presence of sulfated polysaccharides in plants is an adaptation to life in high-salinity environments, which may have been conserved throughout evolution.

To determinate if the presence of sulfated polysaccharides in plants was associated with salt stress conditions, we analyzed plants that were artificially and naturally exposed to various salinities. Experiments with *R. maritima* in an aquarium demonstrated that sulfated galactans completely disappeared when the plant was cultivated in the absence of salt (Fig. 1). The addition of 30 mM magnesium sulfate to plants cultivated in the absence of salt did not induce the biosynthesis of sulfated polysaccharides, indicating that the presence of sulfated polysaccharides in plants is not due to sulfur toxicity. Furthermore, a comparison between the species *R. maritima* and *H. wrightii* collected from environments with different salinities suggested that the sulfated polysaccharide concentration and the degree of sulfation in these compounds are regulated by the salinity in the environment (Fig. 3). Preliminary experiments indicate that the sulfated galactans produced by *R. maritima* have a biosynthetic precursor with lower molecular weight and degree of sulfation (Fig. S2), suggesting that glycosyltransferases and sulfotransferases may function simultaneously during the biosynthesis of sulfated galactans. Because we observed that sulfated polysaccharide biosynthesis in halophytes completely ceases when cultivated in the absence of salt, we speculated as to whether the contrary was possible: Do glycophytes synthesize sulfated polysaccharides in salt-stress conditions? Growth of *O. sativa* in the presence of 200 mM NaCl did not induce the biosynthesis of sulfated polysaccharides, suggesting that their occurrence in plants is an adaptation, rather than an acclimation, to life in high salt environments. In this case, however, we observed a substantial increase in the concentration of carboxylated polysaccharides, suggesting that the presence of negatively charged polysaccharides is important for survival during salt stress (Fig. 4).

The function of sulfated polysaccharides in the plant cell wall in high salt environments remains undetermined. We speculate that the presence of sulfated polysaccharides in the plant cell walls may increase the Donnan potential [18], due to the large negative charge associated with these molecules, which would increase ion density in the vicinity of the plant cell wall, facilitating ion transport at high salt concentrations. A similar mechanism has been proposed for the function of pectin [19], a carboxylated polysaccharide found in the cell wall; we have shown these compounds are also influenced by the concentration of salt in *O. sativa* (Fig. 4).

A survey of halophytes highlights that the occurrence of sulfated polysaccharides in the plant kingdom is more common than previously thought (Fig. 2); all of the halophytes surveyed contained some type of sulfated polysaccharide. The sulfated polysaccharides from the marine angiosperms that we examined are composed of galactose units, while those from mangrove angiosperm species are composed of galactose and arabinose units. Sulfated polysaccharides from the pteridophyte species are composed of glucose units. Interestingly, green algae, the common ancestor of these plants, possessed sulfated polysaccharides composed of the same monosaccharide units: galactose, galactose and arabinogalactose, or glucose units. These results suggest that the genes responsible for the biosynthesis of sulfated polysaccharides may have been conserved throughout plant evolution. Possibly, evolutionary modifications occurred through the activation and inhibition of glycosyltransferase genes, altering the composition of sulfated polysaccharides among the different phyla. Unfortunately, the biosynthesis of sulfated polysaccharides remains unstudied, restricting any further genetic evolutionary analysis.

In conclusion, we suggest that the production of sulfated polysaccharides by higher plants is an adaptation that allows these plants to survive in high-salt environments, which may have been conserved during their evolution. The possible maintenance of the genes for the biosynthesis of these polysaccharides, and their occurrence in halophytes from different evolutionary periods, prompts an interesting insight in regard to atavisms: “An organism's past not only constrains its future; it also provides as legacy an enormous reservoir of potential for rapid morphological change based upon small genetic alterations” [20]. If the genes involved in the biosynthesis of sulfated polysaccharides were conserved during the evolution of halophytic plants, it is conceivable that their expression can be induced in glycophytes; possibly engineering plants with a new mechanism of salt resistance: the presence of sulfated polysaccharides in their cell walls.

## Methods

### Plant samples

Plant specimens were collected from different locations in Brazil, washed with distillated water and sun dried. The following is a list of locations, local salinity, and species collected: (a) “Ilha do Japones”, Rio de Janeiro, Brazil (22°53′S, 42°00′W), 35 ppt, *R. maritima*, *H. wrightii*, and *A. shaueriana*; (b) “Praia da Urca”, Rio de Janeiro, Brazil (22°54′S, 43°10′W), 35 ppt, *H. decipiens*; (c) Cabure, Maranhao, Brazil (2°33′S, 42°43′W), 35 ppt, *R. mangle*; (d) Macau, Rio Grande do Norte, Brazil (5°6′S, 36°38′W), 38 ppt, *H. wrightii*. The glycophyte species (*Z. mays*, *P. vulgaris*, and *O. sativa*) were grown from seeds in the laboratory.

*R. maritima* collected from an environment with a 35 ppt salinity was used for experiments involving varying salinities. Individual ramets and sediment were collected, placed in bins, and immediately transported to the laboratory, where 30 individual ramets (approximately 10 cm in length) were shaken clean in an artificial seawater (ASW) bath. The ramets were planted in aquariums holding 3 L of the collected and sieved sediment and 40 L of ASW (35 ppt salinity). After two weeks, samples were collected and the salinity was gradually decreased over a two-week period until no salt remained (salt concentrations under 0.5 ppt, comparable to freshwater concentrations). The ramets were maintained in the salt-free environment for another two weeks, whereupon more samples were collected. No detectable decrease in growth was observed when *R. maritima* was cultivated in the absence of salt. Finally, for two weeks the salinity was gradually restored by the addition of NaCl (35 ppt salinity), and the samples were collected after two more weeks. Salinity was measured by conductivity.

In a different experiment, rice seeds (*O. sativa*) were allowed to germinate for 15 days in the dark in the presence and absence of 200 mM NaCl (a salinity comparable to ∼12 ppt). Root and shoot lengths decreased about 30% in plants grown under salt stress when compared to the control samples. Samples from each experimental set were collected and processed as described below.

### Extraction and quantification of sulfated and carboxylated polysaccharides

Plant samples were sun-dried and ground in a blender. The triturated samples were incubated two times with 50 mL/g of dry tissue and ethanol or acetone to remove pigments and lipids, respectively. Ten grams from each dry, powdered sample were used for protein digestion with 1 g of papain (Merck, Darmstadt, Germany) in 400 mL 0.1 M sodium acetate (pH 6.0) with 5 mM ethylenediamine tetra-acetic acid, and 5 mM cysteine. After incubating for 24 h at 60°C, the mixture was centrifuged (2500 x g for 30 min), and the supernatant subjected to ethanol precipitation. The pellet was used to conduct a second extraction procedure, and the sulfated polysaccharides were pooled. The procedure was repeated a third time; however, no sulfated polysaccharides were obtained. The sulfated polysaccharides in solution (supernatant) were precipitated with 800 mL absolute ethanol (2 volumes) at 4°C for 24 h. The precipitate was collected by centrifugation (2500 x *g* for 30 min at 4°C), dried in vacuum, and resuspended in distilled water.

### Chemical analysis

The metachromatic assay [21] was used to analyze sulfated polysaccharide concentration, the carbazol reaction [22], [23] to quantify hexuronic acid concentration, and phenol-sulfuric acid reaction [24] was used to measure the total hexose concentration. Sulfate from the sulfated polysaccharides polymers was measured using the barium assay (BaCl_2_/gelatin method) [25].

The monosaccharide composition of sulfated polysaccharides was analyzed by paper chromatography. After acid hydrolysis (6.0 M trifluoroacetic acid, 100°C for 5 h) of the sulfated polysaccharides, monosaccharides were identified by paper chromatography in n-butanol:pyridine:water (3∶2∶1,v/v) for 48 h on Whatman No. 1 paper, followed by staining with silver nitrate. The monosaccharide units were confirmed by gas-liquid chromatography of the derived alditol acetates [26].

### Size exclusion chromatography

The polysaccharides extracted from artificially cultivated *R. maritima* (see above) were fractionated by size exclusion chromatography on a Superose 12 column (16/50; Amersham Pharmacia Biotech, Little Chalfont, UK). The column was equilibrated with 0.2 M sodium bicarbonate (pH 6.0) and eluted with the same solution. The flow rate through the column was 0.5 mL/min, and fractions of 0.5 mL were collected in regular intervals. Fractions were subjected to metachromasy, carbazol, and phenol-sulfuric acid reaction to analyze the sulfated polysaccharide, carboxylated polysaccharide, and total hexose contents, respectively.

### Anion exchange chromatography

Sulfated polysaccharides were purified using anion exchange chromatography with DEAE-cellulose and Mono-Q HR5/5 columns (Amersham Pharmacia Biotech, Little Chalfont, UK). Polysaccharide samples (100 mg) were applied to a DEAE-cellulose column (40 ×2.5 cm), equilibrated with 0.01 M sodium acetate (pH 5.0), and eluted by with a linear gradient of 1–3 M NaCl in the same solution. The flow rate of the column was 5 mL/h, and fractions of 2.0 mL were collected in regular intervals. Fractions were analyzed for sulfated polysaccharides using metachromasy, pooled, dialyzed against distilled water, and lyophilized. The sulfated polysaccharide samples obtained from the DEAE-cellulose column (10 mg) were applied to a MonoQ column coupled with an FPLC system, equilibrated with 20 mM Tris–HCl (pH 8.0) containing 10 mM ethylenediamine tetra-acetic acid. The column was eluted with a linear salt gradient of 0–4 M NaCl in the same solution, and 0.5 ml fractions were collected and assayed for metachromasy. The fractions containing sulfated polysaccharides were pooled, dialyzed against distilled water, and lyophilized.

Agarose gel electrophoresis was performed with ∼10 µg of each sample. Samples were applied to a 0.5% agarose gel in 50 mM 1,3-diaminopropane:acetate (pH 9.0), run at 110 V for 1 h, fixed with 0.1% *N*-cetyl,*N*,*N*,*N*-trimethylammonium bromide solution overnight, dried and stained with 0.1% toluidine blue in acetic acid:ethanol:water (0.1∶5∶5,v/v).

## Supporting Information

Figure S1 **The presence of sulfated galactans in** ***R. maritima*** **is abolished when cultivated in the absence of salt.** Polysaccharides extracted from *R. maritima* cultivated at 35 ppt salinity (1), both after the salt was removed (2), and after the salt was restored (3), were analyzed by agarose gel electrophoresis (A). The sulfate concentration was detected using the barium assay (B).
(TIF)

Figure S2 ***R. maritima*** **utilizes a precursor with a lower degree of sulfation during the biosynthesis of sulfated polysaccharides.** Samples of sulfated galactan from *R. maritima* after the salinity was restored (open circle), after ramets were incubated with artificial seawater (480 mM NaCl_2_, 10 mM KCl, 27 mM MgCl_2_, 10 mM CaCl_2_, and 2 mM NaHCO_3_) in the presence of 40 µCi/mL of Na_2_^35^SO_4_ (solid triangle), were compared to the control (solid circle) using anion-exchange chromatography with a MonoQ-FPLC column and analyzed for their metachromatic properties (A). Additionally, *R. maritima* ramets were incubated with artificial seawater (480 mM NaCl_2_, 10 mM KCl, 27 mM MgCl_2_, 10 mM CaCl_2_, and 2 mM NaHCO_3_) in the presence of 40 µCi/mL of Na_2_^35^SO_4_, and sulfated polysaccharides were extracted and ran on a MonoQ-FPLC column (solid triangle). Fractions from the Na_2_^35^SO_4_ incubated *R. maritima* sample were further analyzed by agarose gel electrophoresis (B), followed by autoradiography (C).
(TIF)

Figure S3 **^1^H NMR spectra at 600 MHz of (A)** ***H. wrightii*** **, (B)** ***H. decipiens*** **, (C)** ***A. shaueriana*** **, (D)** ***R. mangle*** **, and (E)** ***A. aureum*** **.** The spectra were recorded at 60°C for samples in D_2_O solution. The residual water signal was suppressed by presaturation. Chemical shifts are relative to external trimethylsilylpropionic acid at 0 ppm.
(TIF)
