# Supplementary figures and images for: Rising from the Sea: Correlations between Sulfated Polysaccharides and Salinity in Plants
Source: PLoS One. 2011 Apr 28;6(4):e18862. doi: 10.1371/journal.pone.0018862 (PMC3084243; doi:10.1371/journal.pone.0018862)

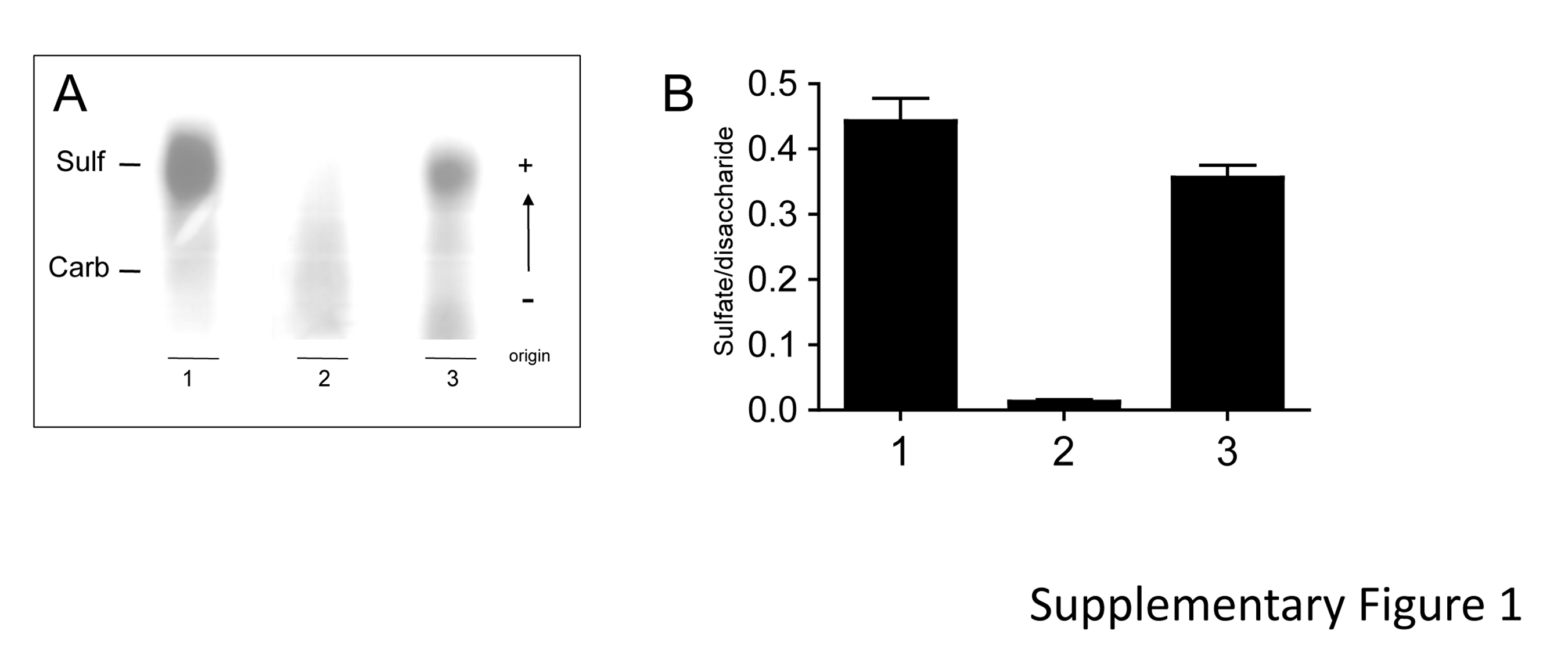

Supplement: Figure S1 — The presence of sulfated galactans in R. maritima is abolished when cultivated in the absence of salt. Polysaccharides extracted from R. maritima cultivated at 35 ppt salinity (1), both after the salt was removed (2), and after the salt was restored (3), were analyzed by agarose gel electrophoresis (A). The sulfate concentration was detected using the barium assay (B). (TIF) [file pone.0018862.s001.tif]

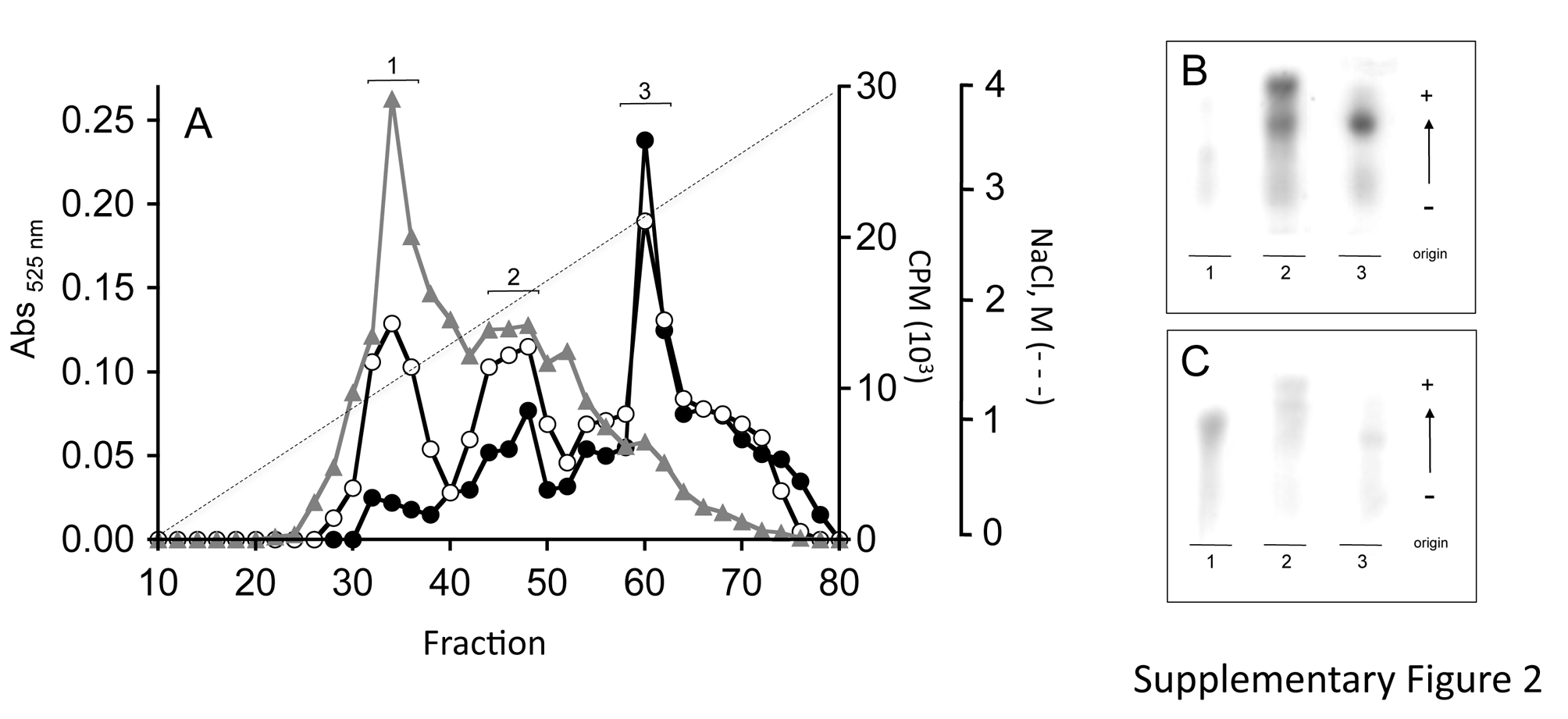

Supplement: Figure S2 — R. maritima utilizes a precursor with a lower degree of sulfation during the biosynthesis of sulfated polysaccharides. Samples of sulfated galactan from R. maritima after the salinity was restored (open circle), after ramets were incubated with artificial seawater (480 mM NaCl2, 10 mM KCl, 27 mM MgCl2, 10 mM CaCl2, and 2 mM NaHCO3) in the presence of 40 µCi/mL of Na2 35SO4 (solid triangle), were compared to the control (solid circle) using anion-exchange chromatography with a MonoQ-FPLC column and analyzed for their metachromatic properties (A). Additionally, R. maritima ramets were incubated with artificial seawater (480 mM NaCl2, 10 mM KCl, 27 mM MgCl2, 10 mM CaCl2, and 2 mM NaHCO3) in the presence of 40 µCi/mL of Na2 35SO4, and sulfated polysaccharides were extracted and ran on a MonoQ-FPLC column (solid triangle). Fractions from the Na2 35SO4 incubated R. maritima sample were further analyzed by agarose gel electrophoresis (B), followed by autoradiography (C). (TIF) [file pone.0018862.s002.tif]

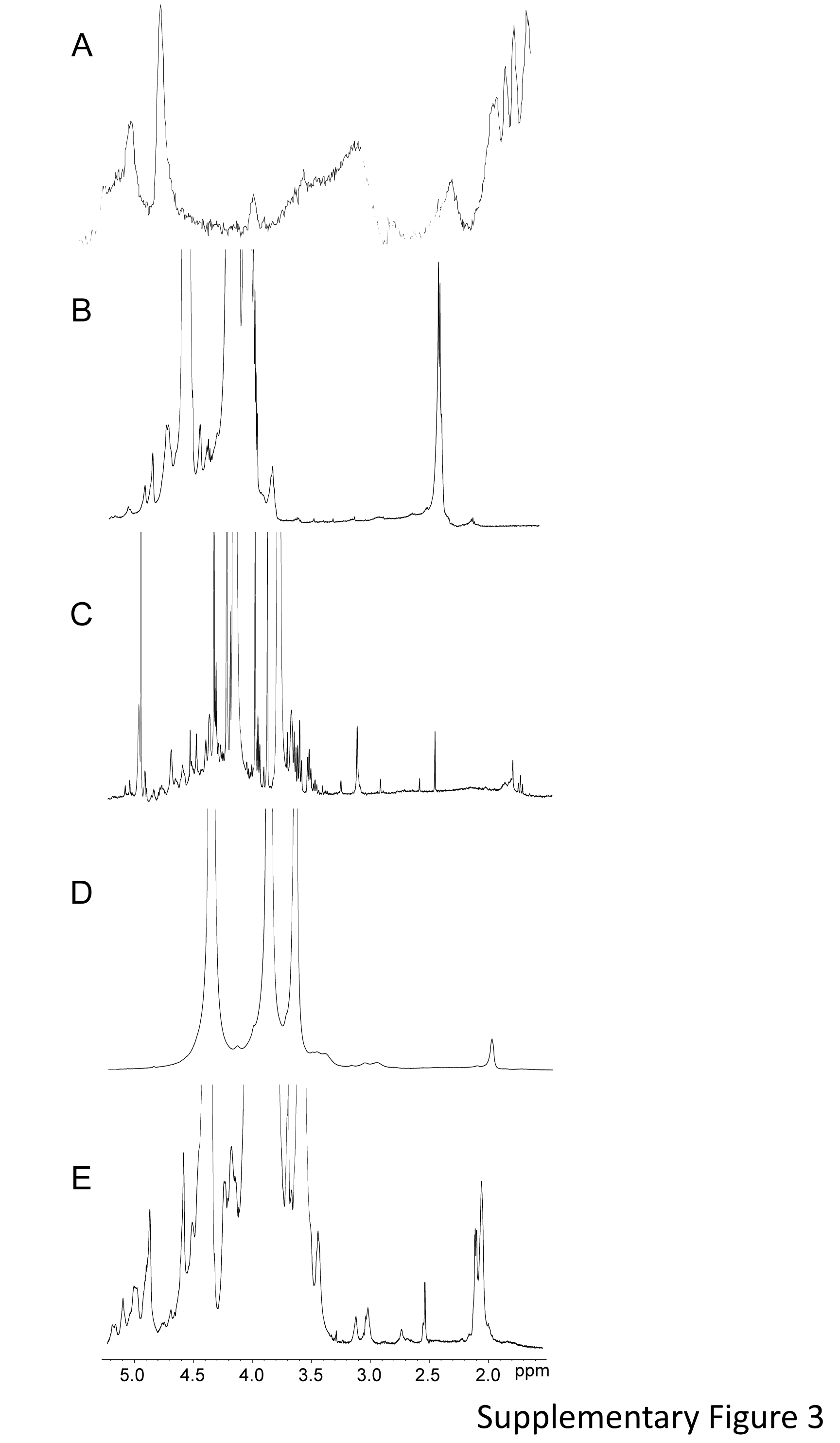

Supplement: Figure S3 — 1H NMR spectra at 600 MHz of (A) H. wrightii , (B) H. decipiens , (C) A. shaueriana , (D) R. mangle , and (E) A. aureum . The spectra were recorded at 60°C for samples in D2O solution. The residual water signal was suppressed by presaturation. Chemical shifts are relative to external trimethylsilylpropionic acid at 0 ppm. (TIF) [file pone.0018862.s003.tif]
